# Odontogenic Myxoma of the Mandible

**DOI:** 10.1155/2012/214704

**Published:** 2012-07-09

**Authors:** Rakesh Kumar Manne, Venkata suneel Kumar, P. Venkata Sarath, Lavanya Anumula, Sridhar Mundlapudi, Rambabu Tanikonda

**Affiliations:** ^1^Department of Oral Medicine and Radiology, Narayana Dental College and Hospital, Chintareddypalem, Nellore 524003, Andhra Pradesh, India; ^2^Department of Conservative Dentistry and Endodontics, R.I.M.S, Government Dental College, Kadapa 516004, Andhra Pradesh, India; ^3^Department of Conservative Dentistry and Endodontics, Narayana Dental College and Hospital, Chintareddypalem, Nellore 524003, Andhra Pradesh, India; ^4^Department of Conservative Dentistry and Endodontics, Vishnu Dental College, Bhimavaram 534202, Andhra Pradesh, India

## Abstract

Odontogenic myxomas are benign but locally aggressive neoplasms found almost exclusively in the jaws and arise only occasionally in other bones. We present a rare case of odontogenic myxoma occurring in the mandible of a 19-year-old male patient with a brief review of clinical and radiological features, and diagnostic and operative dilemmas in managing the same.

## 1. Introduction

Odontogenic myxomas (OMs) are benign tumors derived from embryonic mesenchymal elements of dental anlage [1, 2]. OM appears to originate from dental papilla, follicle, or periodontal ligament. The evidence for its odontogenic origin arises from its almost exclusive location in the tooth bearing areas of the jaws, its occasional association with missing or unerupted teeth, and the presence of odontogenic epithelium [1, 3]. According to the World Health Organization (WHO), OM is classified as benign tumor of ectomesenchymal origin with or without odontogenic epithelium [1]. The odontogenic nature of the myxomas has been challenged by some authors because of the appearances, whilst consistent with odontogenic ectomesenchyme, could also represent a more primitive fibroblastic or undifferentiated tissue [4].

Most of the OMs reported were young adults affected mostly in their second and third decade of life with marked female predilection [1, 3, 5]. OM can occur both in bone and soft tissue. Although intraosseous myxoma has been reported in various anatomical sites, the majority of these tumors occur in the mandible, followed by the maxilla [1, 2, 5, 6]. Clinically, OMs are slow-growing, painless, and site-aggressive tumors. Since pain and hypoesthesia are not common, the lesions may reach a considerable size before patient perceives its existence and seeks treatment. Larger lesions may cause tooth displacement and cortical bone expansion [4, 6]. Radiologically, the appearance may vary from a unilocular radiolucency to a multicystic lesion with well-defined or diffused margins with fine, bony trabeculae within its interior structure expressing a “honey coumbed,” “soap bubble,” or “tennis racket” appearance [1, 7]. A unilocular appearance may be seen more commonly in children and in anterior parts of the jaws. Root resorption is rarely seen, and the tumor is often scalloped between the roots [6].

OMs are not encapsulated, thus promoting significant infiltration into the adjacent medullar bone [4]. The OM exhibits abundant extracellular production of ground substance and thin fibrils by the delicate spindle-shaped cells. These undifferentiated mesenchymal cells are capable of fibroblastic differentiation also. Depending upon the pattern of differentiation, the histological nature of the tumor varies. It may be completely myxomatous tissue or varying proportions of myxomatous and fibrous tissue [1, 4]. Some regard OM as a modified form of fibroma in which myxoid intracellular substance separates the connective tissue [1, 8]. The treatment of choice for OM is surgical excision by enucleation, curettage, or block resection. OM carries a high recurrence rate. Due to poor followup and lack of reports, a precise and accurate recurrence rate is still missing. The high recurrence rate of 25% is reported when more conservative treatments are used [9]. In view of its rarity, large size involving body and ramus of the mandible, and diagnostic and operative dilemmas encountered while managing, the present case is herewith reported.

## 2. Case Report

**Figure 1:**
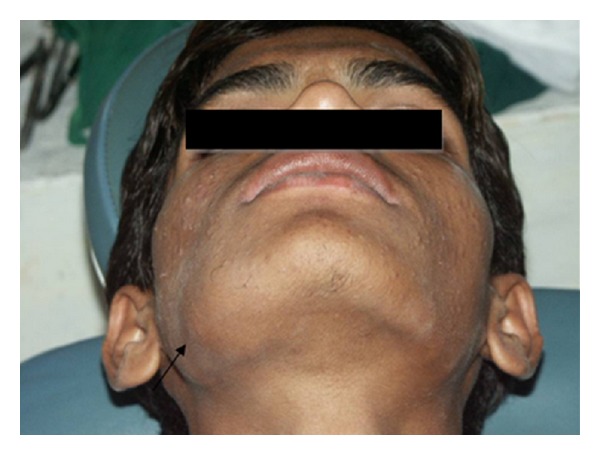
Extraoral photograph, showing swelling in the right-side mandibular body.

**Figure 2:**
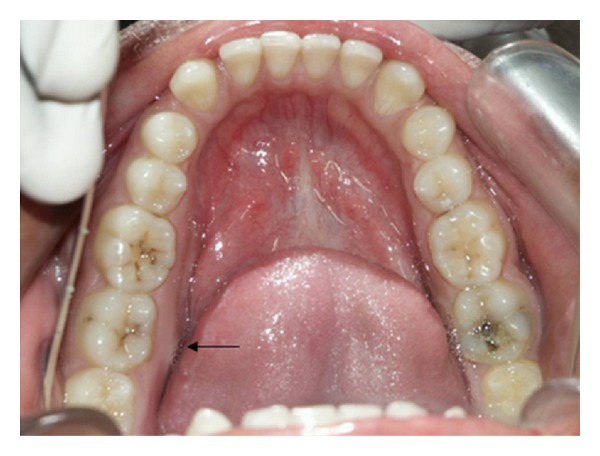
Intraoral photograph, showing slight lingual cortical expansion in the right-side mandibular.

A 19-year-old male patient was referred to the Department of Oral Medicine and Radiology for treatment. Patient gave a one-month history of a mild pain and swelling in the right posterior mandible. Pain is intermediate and usually seen on mastication. Initially, the swelling was small in size and showed a gradual increase to its present dimensions. Clinical examination revealed a firm, non-tender swelling expanding the buccal and lingual cortices of the mandible, extending from right first premolar region to third molar region, and it obliterated the buccal vestibule. The skin over the swelling was normal, and there was no history of paresthesia (Figures 1 and 2).

**Figure 3:**
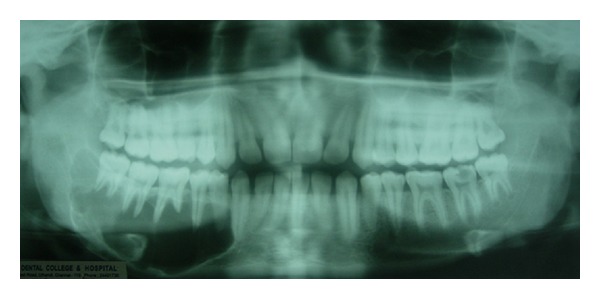
Panoramic radiograph showed a large well defined, sclerotic margined, multilocular radiolucent lesion with “soap bubble” appearance on right side mandible.

**Figure 4:**
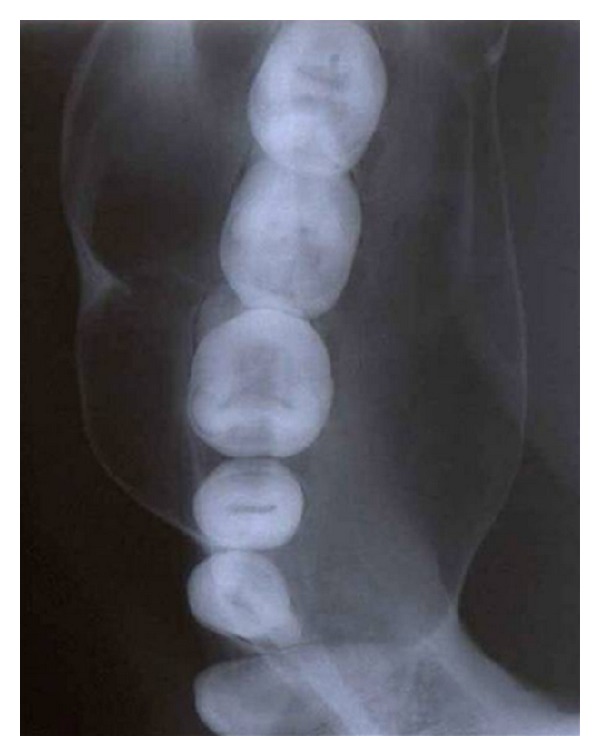
Lateral occlusal radiograph of right side mandible showing expansion of buccal and lingual cortices.

**Figure 5:**
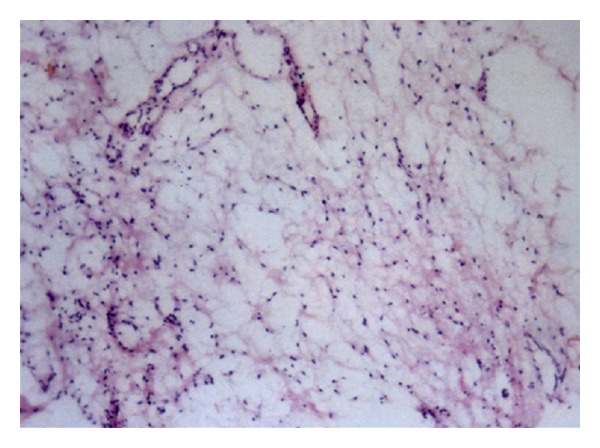
Microphotograph showing mixed area of fibrous tissue and inconspicuous stands of odontogenic epithelium in a myxoid stroma; magnification: ×40.

**Figure 6:**
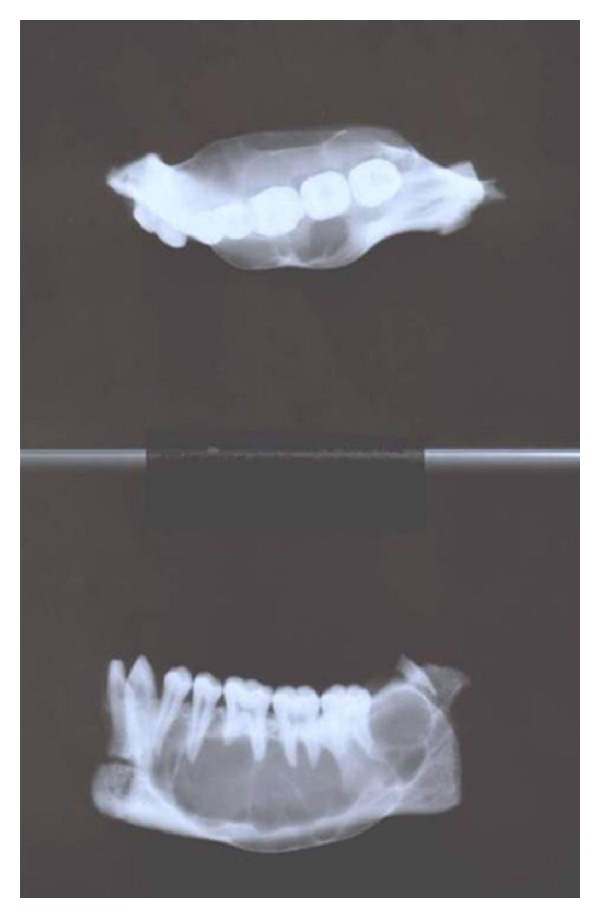
Radiograph of resected segment of right-side mandible.

**Figure 7:**
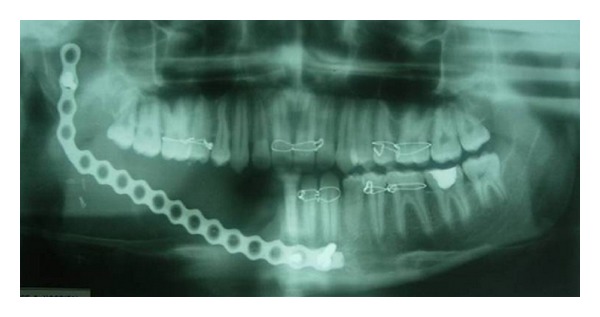
Postoperative panoramic radiograph showing reconstruction of the resected site with microvascular iliac bone grafting and fixation with titanium plate.

The panoramic radiograph showed a large well-defined, sclerotic margined, multilocular radiolucent lesion with “soap bubble” appearance extending from the lower right canine to 1 cm distal to the third molar and also showed first molar mesial root resorption (Figure 3). The right mandibular lateral occlusal radiograph showed multilocular radiolucent lesion with expansions of buccal and lingual cortices (Figure 4). Fine needle aspiration was performed to rule out odontogenic cysts, and results were negative. Benign odontogenic tumors were considered, and incisional biopsy was made and a histopathological examination of the tissue sample exhibited rounded, stellate, and spindle-shaped mesenchymal cells arranged in a loose, myxoid stroma with few collagen fibrils (Figure 5). These results were suggestive of OM. Segmental resection of the right side mandible was performed under general anesthesia (Figure 6). Reconstruction was done by microvascular iliac bone grafting, and fixation was achieved with titanium plates (Figure 7). Postoperative complications included iliac bone graft rejection, and sequestrated bone graft was removed 3 months later. 30 months after the surgical procedure, there were no radiographic or clinical signs of recurrence and patient was not interested for rehabilitation.

## 3. Discussion

The prevalence of OM is principally quoted between 0.04% and 3.7% [10]. In Asia, Europe, and America, relative frequencies between 0.5% and 17.7% have been reported [11]. There was lack of uniformity in the most common age group studies of OM, but most of the studies showed 22.7 to 36.9 years, and it is rarely seen in patients younger than 10 years of age or older than 50 [1, 12]. The mandible appears to be more frequently affected than the maxilla [1, 2, 5, 6]. Our case presented at the age of 19 years involving posterior mandible, which is almost in conformity with the reported literature. There are no clinical or radiological signs that would allow a physician to distinguish myxoma from odontogenic and nonodontogenic lesions; however, histological analysis shows several lesions that could be misinterpreted as myxoma [9]. The majority of the myxomas are almost always asymptomatic, although some patients present with progressive pain in lesions invading into surrounding structures with eventual neurological disturbances [13]. OM of the maxilla is less frequent but behaves more aggressively than that of the mandible [14, 15]. The present case is invading with intermediate pain, and more aggressive, in spite of its mandibular occurrence. OMs radiographically appear as multilocular or unilocular radiolucencies. The present case showed multilocular radiolucency with “soap bubble” appearance. Differential diagnosis like ameloblastoma, ameloblastic fibroma, odontogenic fibroma, central hemangioma, or odontogenic keratocyst along with OM could be listed as initial diagnostic hypothesis based on the clinical and radiological findings [5, 9].

The aggressive nature of OM is well documented in the literature. The tumor is not radiosensitive, and the surgery is the treatment of choice [1, 3, 6, 9]. Questions have been raised in regard with the type of surgical treatment modality that should be applied to each case due to its high recurrence rate [9]. The lack of capsule and infiltrative growth pattern is responsible for high rate of recurrence when conservative treatments like enucleation, curettage, and cryotherapy are performed. The conservative treatments have several advantages over more radical treatment, which would consist of mandibular reconstruction with fibular microsurgical flap. The treatment performed represented a less morbid intervention, the possibility of intraoral access, the absence of donor area, a shorter hospitalization time, not interfering with facial growth in children, and a low procedural cost [16]. Radical treatment of block resection is advised by most authors over conservative treatment due to its invasive nature, large size, and recurrence history even though this intervention poses patients posttreatment rehabilitation difficulties, and we also agreed with most of the authors and treated with block resection for the present case [1, 6, 7, 17]. The conservative management of myxoma by excision and curettage with liquid nitrogen cryotherapy is an alternative method proposed to radical resection. Liquid nitrogen will eliminate any remaining neoplastic cells by bone devitalization without affecting the inorganic structure, thereby yielding new bone formation [18]. A minimum of five years of surveillance is required to confirm that the lesion has healed, and periodical clinical and radiographic followup should be maintained indefinitely irrespective of treatment modality applied to treat OM [19].
